# Computational Modeling of Cellulose Synthase Heterotrimer Assembly and Identification of Antimicrobial Compounds Targeting Interface Sites in *Phytophthora infestans*

**DOI:** 10.3390/jof12030192

**Published:** 2026-03-07

**Authors:** Biju Vadakkemukadiyil Chellappan, P. R. Shidhi, V. S. Amritha, Sherif Mohamed El-Ganainy, Mohammed A. Almalki

**Affiliations:** 1 Department of Biological Science, College of Science, King Faisal University, Al-Ahsa 31982, Saudi Arabia; malmalki@kfu.edu.sa; 2 Department of Zoology, University of Kerala, Karyavattom, Trivandrum 695581, Kerala, India; 3 Department of Arid Land Agriculture, College of Agricultural and Food Sciences, King Faisal University, P.O. Box 420, Al-Ahsa 31982, Saudi Arabia; salganainy@kfu.edu.sa

**Keywords:** cellulose synthases, pleckstrin homology domain, AlphaFold-multimer, oomycete pathogenesis, glycosyltransferase family 2

## Abstract

*Phytophthora infestans*, a devastating oomycete pathogen responsible for late blight in solanaceous crops, relies on cellulose synthase (CesA) complexes for cell wall biosynthesis and virulence. Unlike plant CesAs that form homomeric trimers, oomycete CesA complexes are hypothesized to assemble as heteromeric units, yet their structural organization remains poorly defined. Here, we employed AlphaFold-Multimer and molecular docking to resolve the structural assembly of the PiCesA1–PiCesA2–PiCesA4 heterotrimer in *P. infestans* and identify potential ligand-binding sites for targeted inhibition. Structural modeling revealed a conserved transmembrane architecture combined with a distinctive cytosolic organization, in which N-terminal pleckstrin homology domains play a central role in heteromeric assembly. AlphaFold-Multimer consistently predicted a stable heterotrimer stabilized by cyclic interactions between pleckstrin homology domains and glycosyltransferase-A domains, forming an extensive interface network that is spatially segregated from the conserved UDP-glucose–binding catalytic core. Structure-guided docking identified potential ligands targeting pleckstrin homology–glycosyltransferase interface regions. Notably, these sites are absent or structurally divergent in plant cellulose synthases, underscoring their potential for pathogen-selective targeting. This work advances mechanistic understanding of cellulose biosynthesis in filamentous pathogens and proposes new avenues for selective disease control in agriculture.

## 1. Introduction

Cellulose synthase (CesA) enzymes are large, membrane-integrated glycosyltransferases that polymerize UDP-glucose into β-1,4-glucan chains, which coalesce into cellulose microfibrils that provide mechanical strength and structural integrity in diverse organisms [1]. The overall CesA domain architecture and the core mechanism of cellulose biosynthesis are broadly conserved from bacteria to land plants. In bacteria, cellulose synthases typically operate as individual catalytic units or within relatively small complexes, whereas in plants they assemble into higher-order triangular homotrimers that act as fundamental building blocks of the larger rosette-type cellulose synthase complexes [2,3]. In filamentous oomycetes, cellulose is a key structural component of the cell wall, distinguishing them from true fungi and highlighting the central role of CesA-mediated cellulose biosynthesis in growth, morphogenesis, and pathogenic development [4]. Although multiple CesA isoforms have been identified across oomycete genomes, the molecular organization, assembly mechanisms, and regulatory control of oomycete CesA complexes remain largely undefined at the structural level [5,6,7].

*Phytophthora infestans*, the causal agent of potato and tomato late blight, is one of the most destructive plant pathogens worldwide, posing a persistent threat to global food security [8]. Genomic analyses of *P. infestans* have identified four CesA isoforms (PiCesA1, PiCesA2, PiCesA3 and PiCesA4), all containing conserved glycosyltransferase family 2 (GT2) catalytic domains but differing in their auxiliary domains [7,9]. Recent biochemical analyses in *Phytophthora capsici* demonstrated that PcCesA1, PcCesA2, and PcCesA4 co-immunoprecipitated as constituents of a multiprotein complex, whereas PcCesA3 exhibits markedly weaker association or no association. Notably, both detergent-solubilized membrane extracts and recombinant PcCesA1 catalyze exclusively the formation of short-chain soluble β-1,4-glucans (predominantly cellobiose), rather than cellulose microfibrils, underscoring the requirement for intact heteromeric complex formation for productive cellulose biosynthesis. Given the high sequence conservation, shared domain architecture, and similar transmembrane topology between CesAs from *P. capsici* and *P. infestans*, these findings strongly suggest that a comparable heterotrimeric CesA1-CesA2-CesA4 complex operates in *P. infestans* [6]. However, the structural basis of this assembly and its vulnerability to chemical disruption remain largely unexplored.

The advent of artificial intelligence (AI)-based protein structure prediction has revolutionized macromolecular structural biology [10,11]. AlphaFold3 has demonstrated near-experimental accuracy in predicting monomeric protein structures, while AlphaFold-Multimer extends these capabilities to multimeric protein complexes, including large and asymmetric heteromeric assemblies [12,13]. Beyond generating high-confidence structural models, AlphaFold-Multimer provides confidence metrics such as the predicted aligned error (PAE), enabling quantitative evaluation of inter-subunit positioning and interface reliability [14,15]. In plant–pathogen interacting systems, AlphaFold-Multimer has revealed novel protein–protein interactions that were later validated experimentally, underscoring the broad relevance of AI-driven approaches in modern structural biology [16,17].

*Bacillus* species are widely recognized as effective biocontrol agents against plant pathogens, including oomycetes, due to their ability to produce diverse secondary metabolites with antimicrobial and plant-protective activities [18]. These compounds have been shown to interfere with pathogen growth, cell wall integrity, and virulence processes through multiple mechanisms [19]. Their structural diversity and established biological relevance make *Bacillus*-derived metabolites attractive candidates for exploring new inhibitory strategies targeting pathogen-specific molecular machinery such as cellulose synthase complexes [20].

In this study, we employed AlphaFold-Multimer to model the heterotrimeric assembly of PiCesA1, PiCesA2, and PiCesA4 from *P. infestans*, characterizing its domain architecture and inter-subunit interaction network. The predicted complex was independently evaluated using protein–protein docking and molecular dynamics (MD) simulations to assess interface stability and conformational robustness. Furthermore, structure-based molecular docking was conducted to explore ligand binding at key functional sites within the CesA complex, using a curated library of natural compounds derived from *Bacillus* species. By integrating AI-driven structural modeling with docking and MD simulations, this work provides mechanistic insights into CesA assembly and highlights potential strategies for targeting essential protein–protein interfaces in oomycete cellulose biosynthesis.

## 2. Materials and Methods

### 2.1. Retrieval of PiCesA Sequences and Phylogenetic Analysis

Protein sequences of *P. infestans* cellulose synthases PiCesA1, PiCesA2, PiCesA3, PiCesA4 (Table S1) were retrieved in FASTA format from the UniProt database (https://www.uniprot.org, accessed on 12 October 2025). Homologous CesA sequences from representative *Phytophthora* species and related oomycetes, along with selected plant CesA homologs, were collected to assess evolutionary relationships and isoform divergence (Table S2). Multiple sequence alignment was performed using MUSCLE implemented in MEGA 12.1 [21]. Phylogenetic analysis was conducted in MEGA 12.1 using the Maximum Likelihood method with the Jones–Taylor–Thornton (JTT) substitution model, and branch support was evaluated by 1000 bootstrap replicates [22]. The resulting phylogenetic tree was visualized and annotated using the Interactive Tree of Life (iTOL) web server [23].

### 2.2. Structural Modeling and Validation of PiCesA Monomers

Three-dimensional structures of PiCesA1, PiCesA2, and PiCesA4 were predicted using AlphaFold3. Model confidence was assessed using AlphaFold-derived metrics, including per-residue predicted Local Distance Difference Test (pLDDT) scores and predicted aligned error (PAE) profiles [15,24]. Structural stereochemical quality was further evaluated using PROCHECK Ramachandran plot analysis and ERRAT2 to assess backbone geometry and non-bonded interaction quality [25,26]. Prior to downstream analyses, all predicted structures were subjected to restrained energy minimization using the CHARMM force field to relieve steric clashes and optimize side-chain conformations while preserving the overall fold [27].

### 2.3. Prediction and Validation of the PiCesA1-PiCesA2-PiCesA4 Heterotrimer

The PiCesA1–PiCesA2–PiCesA4 heterotrimer was predicted using the AlphaFold2 Multimer v3 model implemented through ColabFold v1.5.5. Structural templates were not used during prediction. Multiple sequence alignments were generated using the MMseqs2 pipeline with UniRef and environmental sequence databases, and unpaired sequence matching was enabled using a greedy pairing strategy. Sequence depth was limited to 508 clustered sequences with up to 2048 additional sequences [12]. Five models were generated, and the final assembly was selected based on low inter-subunit predicted aligned error (PAE), consistent subunit orientation, proper alignment of transmembrane helices, and absence of steric clashes. Inter-subunit interface residues were identified based on spatial proximity and AlphaFold confidence scores and further analyzed using PDBsum v.1.0.1 to characterize hydrogen bonds, salt bridges, and interface contacts [28].

To further assess the compatibility of the predicted inter-domain interfaces, domain-domain docking was performed using HADDOCK v2.4, with residues corresponding to the predicted interface regions defined as active restraints. Top ranked docked complex was selected using HADDOCK scores, Z-scores, and buried surface area metrics [29]. To validate the predicted heterotrimeric assembly, the top-ranked HADDOCK docked complexes were structurally superimposed onto the PiCesA trimer assembly, enabling geometric comparison of domain orientations and interface positioning.

### 2.4. Ligand Preparation and Molecular Docking

A curated library of *Bacillus*-derived secondary metabolites was compiled for docking. Ligand structures were obtained from PubChem or generated in ChemDraw v.25 and converted into three-dimensional conformations [30,31]. Ligand preparation, including geometry optimization and energy minimization, was performed using the MMFF94 force field as implemented in the PyRx platform via Open Babel to relieve steric strain and obtain low-energy conformers [32,33]. Protonation states were assigned consistent with physiological pH, and Gasteiger partial charges were added prior to docking.

Molecular docking was performed using AutoDock Vina v.2.0 implemented in the PyRx v. 0.9 [34]. Prepared protein structures were converted to PDBQT format following removal of nonessential molecules and addition of polar hydrogens. Docking grid boxes were defined around interface residues in the GT-A domain of each protein, with the grid center set to (X, Y, Z) = (−3.23, 11.66, 33.71) Å and dimensions of 25 × 25 × 25 Å^3^. Docking was performed with flexible ligands and rigid protein receptors using default AutoDock Vina parameters with an exhaustiveness value of 8. Docked poses were ranked according to predicted binding affinity. The resulting complexes were analyzed using Discovery Studio v24.1.0. Final ligand–protein complexes were selected based on binding affinity, consistency of binding poses, and absence of steric clashes, and were subsequently used for molecular dynamics simulations.

### 2.5. Molecular Dynamics Simulations and Binding Free-Energy Calculations

Molecular dynamics (MD) simulations were performed to evaluate the structural stability of PiCesA monomers, the PiCesA1–PiCesA2–PiCesA4 heterotrimer, and selected protein–ligand complexes. For ligand-bound systems, the lowest-energy docking pose was used as the starting configuration. Simulations were conducted using GROMACS 2025.4 with the CHARMM27 force field and the SPC water model. Each system was placed in a triclinic simulation box, solvated, neutralized with Na^+^ ions, and supplemented with 0.15 M salt to approximate physiological conditions. Energy minimization was carried out using the steepest-descent integrator for 5000 steps to remove steric clashes and optimize geometry. The systems were then equilibrated sequentially under NVT and NPT ensembles at 300 K and 1 bar for 100 ps [35]. Production simulations were subsequently performed using the MD integrator for 100 ns under periodic boundary conditions. Trajectory analyses included root mean square deviation (RMSD), residue-wise root mean square fluctuation (RMSF), and time evolution of hydrogen bonds to assess conformational stability and the persistence of inter-subunit or ligand interactions [36].

## 3. Results

### 3.1. Structural Features of PiCesA Monomers

**Figure 1:**
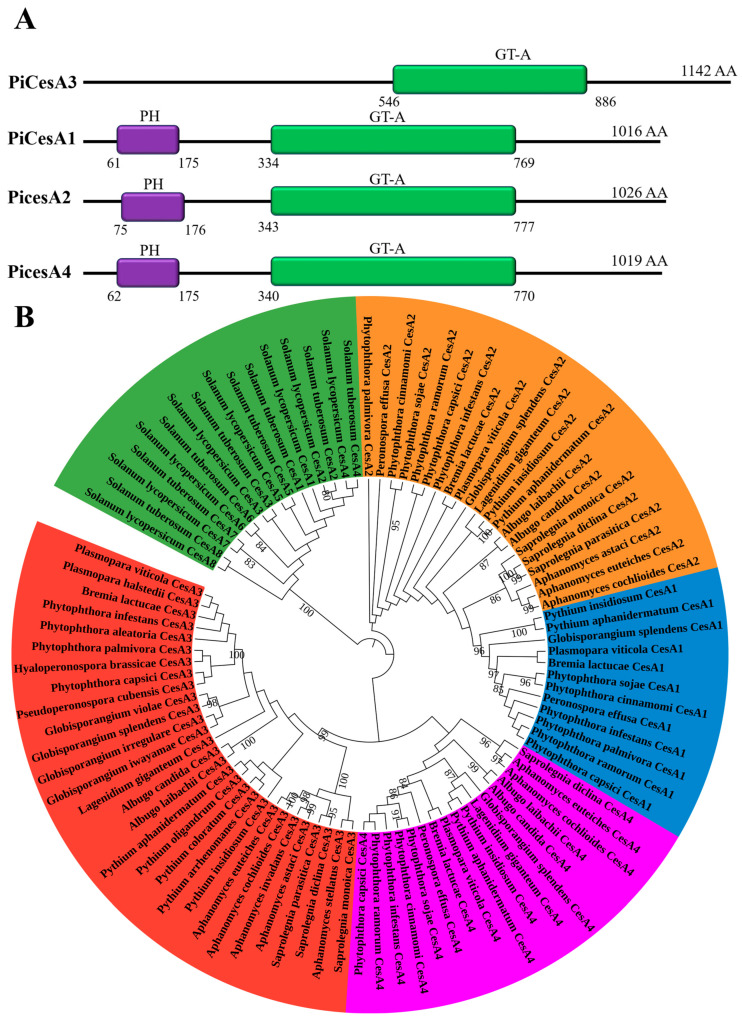
Domain organization and phylogenetic relationships of cellulose synthase (CesA) proteins in *P. infestans* and related oomycetes. (**A**) Schematic domain organization of PiCesA1–PiCesA4 showing the N-terminal pleckstrin homology (PH) domain (purple) and the conserved GT-A catalytic domain (green). PiCesA3 lacks a detectable PH domain. Protein lengths (amino acids) are indicated. (**B**) Circular phylogenetic tree of CesA homologs from *Phytophthora* and related oomycetes, revealing distinct clustering of CesA1–CesA4 paralogs and supporting their evolutionary divergence and functional specialization.

As illustrated in Figure 1, *P. infestans* cellulose synthase isoforms PiCesA1, PiCesA2, and PiCesA4 share a conserved domain architecture featuring an N-terminal pleckstrin homology (PH) domain and a C-terminal GT-A catalytic domain. In contrast, PiCesA3 lacks the PH domain, a feature that is also absent in plant CesAs, highlighting a lineage-specific structural adaptation among oomycete CesAs (Figure 1A). Moreover, phylogenetic analysis across representative oomycetes revealed that PiCesA1, PiCesA2, and PiCesA4 cluster within well-supported, isoform-specific clades conserved across multiple oomycete lineages, while plant CesAs from Solanum spp. formed a clearly distinct and distant lineage (Figure 1B). Notably, within the oomycete clade, CesA1, CesA2, and CesA4 grouped more closely with each other than with CesA3, consistent with biochemical evidence indicating their cooperative involvement in multimeric cellulose synthase complex assembly [6]. Based on this conserved domain organization, evolutionary relationship, and available biochemical evidence, PiCesA1, PiCesA2, and PiCesA4 were selected for structural modeling and subsequent interaction analyses.

**Figure 2:**
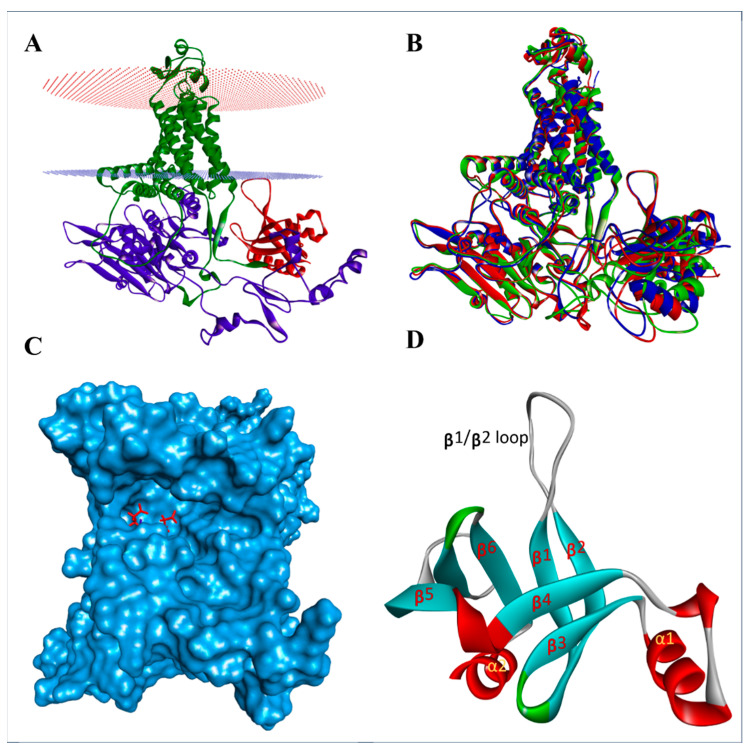
Structural modeling of PiCesA monomers. (**A**) AlphaFold-predicted structure of PiCesA2 shown in cartoon representation. The transmembrane helices are colored green, the GT-A catalytic domain is shown in purple, and the pleckstrin homology (PH) domain is highlighted in red, illustrating the spatial organization of the membrane-embedded region and the cytosolic catalytic domains relative to the membrane planes. (**B**) Structural superposition of PiCesA monomers (PiCesA1, PiCesA2, and PiCesA4) demonstrating a high degree of overall fold conservation. (**C**) Surface representation of the GT-A catalytic domain highlighting the predicted substrate-binding pocket, with key catalytic aspartate residues shown as red sticks. (**D**) Detailed architecture of the pleckstrin homology (PH) domain, illustrating the β-sheet core (β1–β6), α-helices (α1–α2), and the prominent β1/β2 loop implicated in protein–protein interactions.

The AlphaFold-predicted structures of PiCesAs reveal the characteristic cellulose synthase architecture, with seven transmembrane helices forming a compact membrane-anchoring module, while the GT-A catalytic domain and the N-terminal pleckstrin homology (PH) domain extend into the cytosol [37,38]. This organization is consistent with their respective roles in UDP-glucose binding and glucan polymerization, as well as membrane association and spatial regulation of cellulose synthase activity. Structural superposition of PiCesA1, PiCesA2, and PiCesA4 demonstrated a high degree of overall fold conservation among the three isoforms (Figure 2B). The catalytic cores aligned closely, with only minor deviations restricted to flexible loop regions and inter-domain linkers, indicating preservation of a common structural framework required for processive cellulose synthesis. Surface representation of the GT-A catalytic domain revealed a well-defined substrate-binding pocket located within the conserved catalytic cleft, encompassing the hallmark DDDxxQQRR motif essential for UDP-glucose coordination and catalysis (Figure 2C) [39]. The PH domain of PiCesAs adopts a compact β-sandwich fold composed of six antiparallel β-strands (β1–β6) arranged into two nearly perpendicular β-sheets, forming a stable barrel-like core [40]. A prominent and elongated β1/β2 loop protrudes from one edge of the β-sandwich and likely constitutes the principal ligand- or interaction-binding surface, analogous to the phosphoinositide-recognition loops described for canonical PH domains in eukaryotic signaling proteins (Figure 2D) [40]. Flanking the β-sandwich, an N-terminal α-helix (α1) and a C-terminal capping helix (α2) pack against the β-sheet surfaces, reinforcing the hydrophobic core while simultaneously providing exposed interaction interfaces that may mediate contacts with membranes or regulatory partners within the cellulose synthase complex (Figure 2D).

Model confidence was assessed using AlphaFold-derived metrics, including per-residue predicted Local Distance Difference Test (pLDDT) scores and predicted aligned error (PAE) profiles (Table S3, Figures S1 and S2). Across all three PiCesA monomers, the GT-A catalytic and PH domains exhibited consistently high pLDDT values, indicating reliable local geometry and well-defined folding. Independent stereochemical validation using ERRAT2 and PROCHECK further supported model quality, yielding high ERRAT scores of 96.19% (PiCesA1), 95.15% (PiCesA2), and 93.52% (PiCesA4) (Table S3). Ramachandran plot analysis showed that 98.8–99.8% of residues fell within allowed regions across all models, supporting appropriate backbone geometry and stereochemical consistency (Table S3, Figure S3). These high-quality validated monomeric structures provide a robust foundation for subsequent heterotrimeric assembly prediction and structure-based docking analyses.

### 3.2. Trimeric Organization of PiCesA Complex

**Figure 3:**
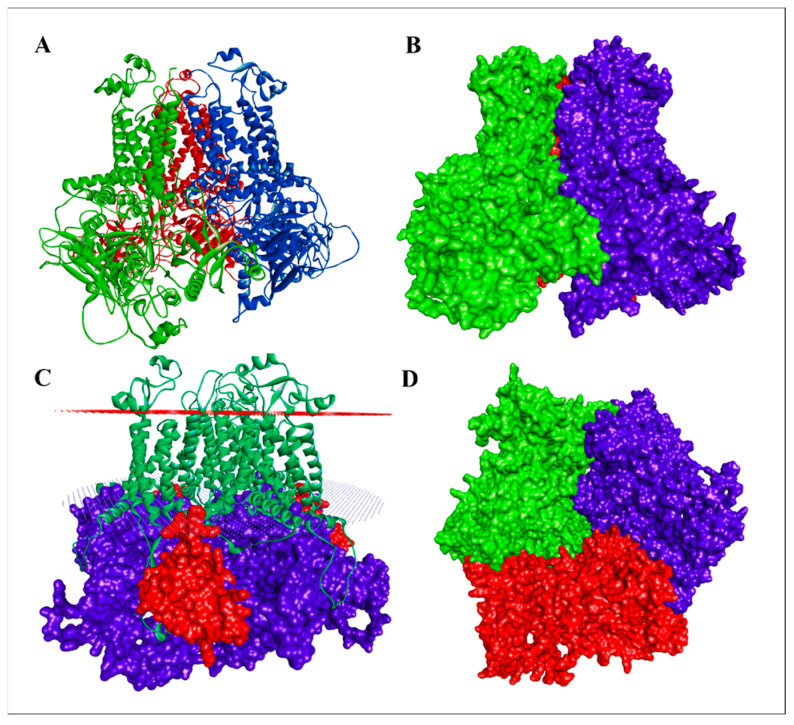
AlphaFold-Multimer prediction of the PiCesA1–PiCesA2–PiCesA4 heterotrimeric assembly. (**A**) Cartoon representation of the predicted PiCesA trimer showing the relative spatial organization of PiCesA1 (green), PiCesA2 (purple), and PiCesA4 (red. (**B**) Surface representation of the PiCesA heterotrimer highlighting the overall assembly architecture with PicesA1 shown in green and PicesA2 shown in purple (**C**) Trimeric complex positioned relative to the membrane planes, illustrating the aligned transmembrane helices forming a membrane-embedded core and extensive cytosolic inter-subunit contacts. (**D**) Bottom surface view of the complete PiCesA heterotrimer, revealing a compact triangular arrangement of the three subunits and the extensive interface regions stabilizing the assembly.

AlphaFold-Multimer predictions revealed a stable heterotrimeric assembly comprising PiCesA1, PiCesA2, and PiCesA4, arranged in a well-defined and reproducible architecture (Figure 3A). The predicted assembly displayed a compatible arrangement of the C-terminal transmembrane helices, forming a compact membrane-embedded core, while the large cytosolic regions extended outward and contributed to extensive inter-subunit contacts (Figure 3B–D). Analysis of predicted aligned error (PAE) matrices revealed low PAE values within individual subunits and across specific inter-subunit regions, supporting confident relative positioning of these domains. Elevated PAE values were largely restricted to terminal segments, consistent with intrinsic conformational flexibility rather than uncertainty in the core architecture. Across independent AlphaFold-Multimer predictions, the overall topology of the PiCesA1-PiCesA2-PiCesA4 complex was highly consistent, with no apparent steric clashes or unfavorable domain overlaps. All inter-domain contacts were localized to the cytosolic side of the membrane, and residues contributing to subunit association corresponded to regions exhibiting high AlphaFold confidence scores in the monomer models, supporting the structural reliability of the predicted multimeric assembly.

### 3.3. Interface Analysis of the PiCesA Trimer Assembly

**Figure 4:**
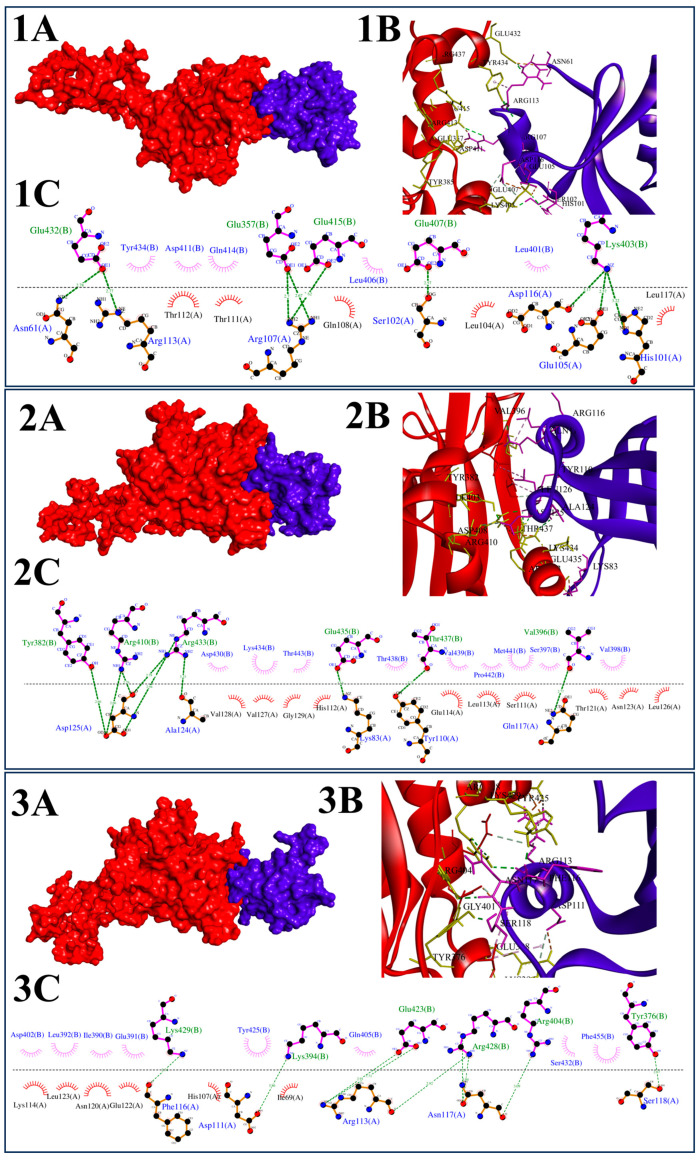
Interface architecture and domain-specific organization within the PiCesA assembly. (**A**–**C**) Inter-subunit interfaces stabilizing the PiCesA heterotrimer. Panel 1 shows the interface between the PH domain of PiCesA1 (purple) and the GT-A domain of PiCesA2 (red): (**1A**) surface representation, (**1B**) cartoon view of domain engagement, and (**1C**) two-dimensional interaction map highlighting hydrogen bonds. Panel 2 depicts the interface between the PH domain of PiCesA2 (purple) and the GT-A domain of PiCesA3 (red), shown as surface (**2A**), cartoon (**2B**), and residue-level interaction network (**2C**). Panel 3 illustrates the interface between the PH domain of PiCesA3 (purple) and the GT-A domain of PiCesA1 (red), with corresponding surface (**3A**), cartoon (**3B**), and interaction schematic (**3C**). These interfaces reveal a conserved PH–GT domain interaction mode that underpins the cyclic and cooperative assembly of the PiCesA heterotrimer.

Detailed inspection of the AlphaFold-Multimer models revealed discrete and well-defined inter-subunit interfaces stabilizing the PiCesA assembly (Figure 4). In all three pairwise interactions viz, PiCesA1-PiCesA2, PiCesA2-PiCesA4, and PiCesA4-PiCesA1, the interfaces were characterized by dense networks of hydrogen bonds and salt bridges involving surface-exposed residues within the cytosolic regions of the proteins. These interactions consistently occurred between the pleckstrin homology (PH) domain of one subunit and the GT-A domain of an adjacent subunit. Mapping of interface residues onto PH-domain structures revealed that the PH-domain contribution to inter-subunit association is predominantly mediated by helix 1 (Alpha 1) (Figure 2D), which forms a rigid, surface-exposed interaction platform. This helix-1-centered interface is spatially distinct from the canonical ligand-binding surface of the PH domain, which is located on the β-sandwich and associated loop regions, indicating functional segregation within the PH domain (Figure 2D).

At the PiCesA1–PiCesA2 interface, residues from helix 1 of the PiCesA1 PH domain, including Glu105, Arg107, Arg113, and Asp116, engaged a lateral surface of the PiCesA2 GT-A domain involving Glu357, Glu415, Glu432, and Lys403. Arg107 and Arg113 acted as interaction hubs, forming multiple short-distance electrostatic contacts with acidic residues on PiCesA2, while Glu105 established reciprocal ionic interactions with Lys403, collectively defining a compact and electrostatically stabilized interface (Figure 4(1A–1C)). The PiCesA2–PiCesA4 interface exhibited a similar interaction pattern dominated by hydrogen bonds and salt bridges. Key contributors included Lys83 and Asp125 of PiCesA2 interacting with Glu435, Arg410, and Arg433 of PiCesA4. Asp125 formed multiple hydrogen bonds and ionic interactions with arginine residues on PiCesA4, whereas Lys83 established strong salt bridges with Glu435, resulting in a tightly interconnected contact surface (Figure 4(2A–2C)). At the PiCesA4–PiCesA1 interface, Arg113 of PiCesA4 forms multiple hydrogen bonds and salt bridges with Glu423 of PiCesA1. Additional stabilizing interactions involved Asn117, Phe116, and Ser118 of PiCesA4 interacting with Arg404, Arg428, Lys429, and Tyr376 of PiCesA1. The clustering of charged residues at this interface highlights a conserved electrostatic strategy for subunit stabilization across the assembly (Figure 4(3A–3C)).

Mapping of all interface residues onto surface representations of the GT-A domains revealed a consistent spatial organization across all three interactions. In each case, the interface occupied a lateral, surface-exposed region of the GT-A domain that was clearly distinct from the predicted substrate-binding pocket, which resides deeper within the conserved catalytic groove (Figure S4). This conserved topological separation indicates that catalytic activity and inter-subunit association are mediated by structurally independent surfaces.

To further evaluate the structural robustness and geometric compatibility of the inter-subunit interfaces predicted by AlphaFold-Multimer, PH–GT-A domain docking was performed using HADDOCK, guided by interface residues identified from the AlphaFold-Multimer models. The top-ranked docking clusters displayed favorable HADDOCK scores and substantial buried surface areas, supporting the structural plausibility and stability of the PH–GT inter-subunit contacts (Table S4). The strong concordance between HADDOCK docking poses and AlphaFold-Multimer assemblies provides independent validation of the proposed PiCesA inter-domain interaction framework (Figure S4).

### 3.4. Molecular Dynamics Simulation of PiCesA Monomers and Heterotrimer Assembly

**Figure 5:**
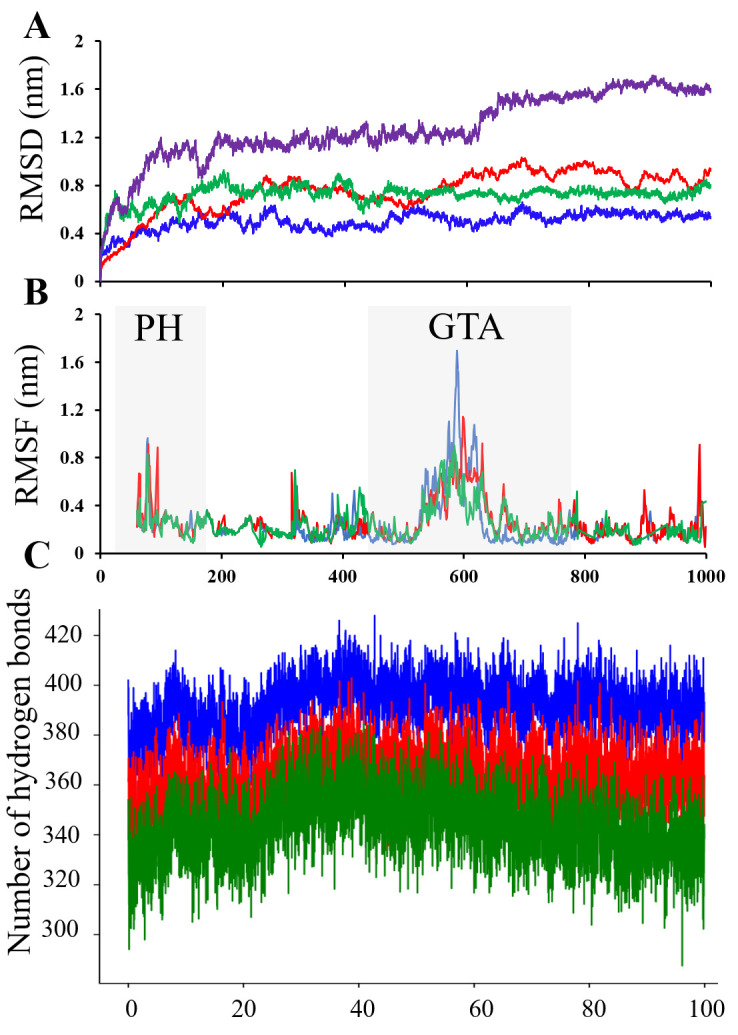
Molecular dynamics simulation of PiCesA monomers and heterotrimer assembly (**A**) Backbone RMSD profiles of PiCesA1 (blue), PiCesA2 (red), PiCesA4 (green), and the heterotrimer (violet), indicating overall structural stability during the simulation. (**B**) RMSF profiles of PiCesA1, PiCesA2, and PiCesA4, highlighting residue-level flexibility, with PH and GT-A catalytic domains indicated. (**C**) Time evolution of hydrogen bonds within the docked PH–GT-A complexes, PiCesA1(PH)-PiCesA2(GT-A), PiCesA2(PH)-PiCesA4(GT-A), and PiCesA4(PH)-PiCesA1(GT-A), reflecting the stability of inter-subunit interactions throughout the simulation.

To assess conformational stability and dynamics, 100 ns all-atom MD simulations were performed for the AlphaFold-predicted PiCesA monomers and the PiCesA1–PiCesA2–PiCesA4 heterotrimer (Figure 5). Backbone RMSD analyses showed rapid equilibration and stable trajectories for all monomers, with PiCesA1 displaying the lowest RMSD (~0.45–0.60 nm) and PiCesA2 and PiCesA4 exhibiting slightly higher but stable plateaus (~0.65–1.0 nm) (Figure 5A). The heterotrimer displayed higher RMSD values (~1.2–1.7 nm), consistent with collective inter-subunit motions rather than structural destabilization, and remained stable throughout the simulation. RMSF analysis revealed low flexibility within the GT-A catalytic cores across all isoforms, whereas higher fluctuations were confined to surface loops, terminal regions, and inter-domain linkers (Figure 5B). The PH domains were similarly stable, with localized flexibility at terminal segments. Analysis of inter-subunit hydrogen bonds demonstrated persistent and stable PH–GT-A interactions across all three interfaces throughout the trajectory (Figure 5C). Together, these results support the dynamic stability of both PiCesA monomers and the heterotrimeric assembly, reinforcing its structural integrity and suitability for structure-based inhibition studies.

### 3.5. Molecular Docking Analysis Targeting the Trimer Interface

**Figure 6:**
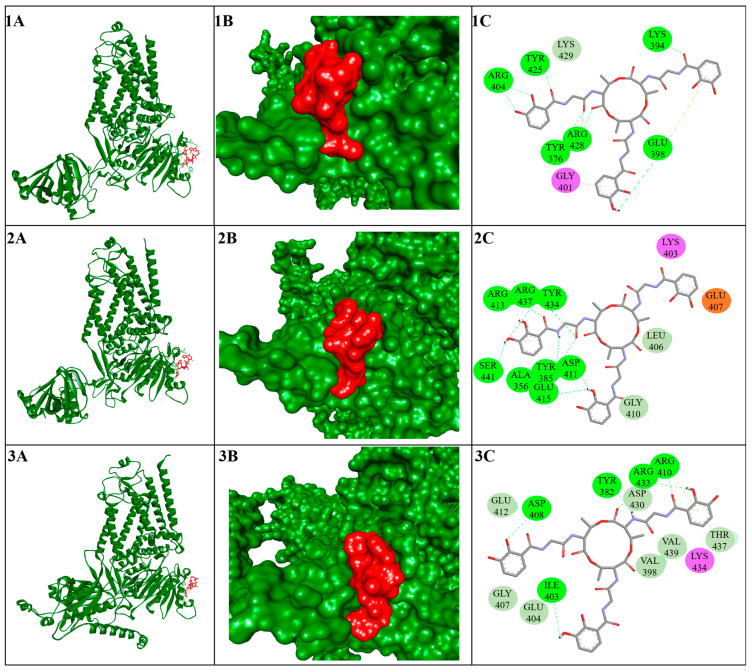
Molecular docking analysis of Bacillibactin binding at the GT-A domain interface of *P. infestans* cellulose synthase (PiCesA) proteins. (**1A**–**3A**) Cartoon representations of Bacillibactin (red sticks) docked at the GT-A interface regions of PiCesA1, PiCesA2, and PiCesA4, respectively. (**1B**–**3B**) Surface representations of the corresponding GT-A domains (green) showing Bacillibactin (red surface) occupying shallow lateral grooves associated with inter-subunit interface regions, spatially distinct from the catalytic site. (**1C**–**3C**) Two-dimensional interaction maps illustrating residue-level contacts between Bacillibactin and GT-A interface residues of PiCesA1, PiCesA2, and PiCesA4. Color code: green dashed lines indicate conventional hydrogen bonds; orange dashed lines represent π–anion or electrostatic interactions; pink/purple dashed lines denote π–alkyl or hydrophobic contacts; light green residue circles indicate polar or charged amino acids, while gray residues denote nonpolar contacts.

To explore the inhibitory potential of *Bacillus*-derived secondary metabolites against *P. infestans* CesA proteins, structure-based docking of Bacillus-derived metabolites was performed at the GT-A inter-subunit interface critical for trimer formation. Molecular docking at the GT-A interface region of PiCesA proteins identified a consistent subset of *Bacillus*-derived secondary metabolites with strong and reproducible binding across PiCesA1, PiCesA2, and PiCesA4 (Table 1). Among the 50 screened compounds, Bacillibactin, Lipoamicoumacin B, Bacillaene, Leodoglucomide A, and Gageotetrin A emerged as top hits, exhibiting binding affinities ranging from −7.4 to −9.6 kcal·mol^−1^. Notably, Bacillibactin displayed the strongest binding across all three isoforms, with the highest affinity observed for PiCesA1 (−9.6 kcal·mol^−1^), followed by PiCesA2 (−9.3 kcal·mol^−1^) and PiCesA4 (−8.9 kcal·mol^−1^), indicating a conserved and energetically favorable interface-recognition mode (Figure 6(1A–3C)).

Interaction mapping revealed that binding is dominated by polar and electrostatic contacts involving conserved arginine, tyrosine, and aspartate residues located on the lateral surface of the GT-A domain, rather than within the catalytic UDP-glucose–binding groove. Across all three PiCesA isoforms, residues such as Tyr376/Tyr382, Arg404/Arg410/Arg433, Asp408/Asp430, and Ser432 repeatedly contributed hydrogen bonds and charge-assisted interactions (Table 1). These residues coincide with the inter-subunit contact surface identified in the AlphaFold-Multimer trimer model, supporting the hypothesis that these ligands act as interface blockers rather than classical competitive inhibitors (Figure 4). Hydrophobic contacts involving Leu, Val, Ile, and Phe residues further stabilized ligand binding by anchoring the elongated aliphatic scaffolds of these compounds within shallow interfacial grooves, a feature particularly evident for bacillaene and lipoamicoumacin B. Moreover, multiple sequence alignments of PiCesA1, PiCesA2, and PiCesA4 orthologs revealed that residues interacting with these ligands are conserved across representative oomycete species (Figures S5–S7), supporting the relevance of this binding surface as a selective target.

**Figure 7:**
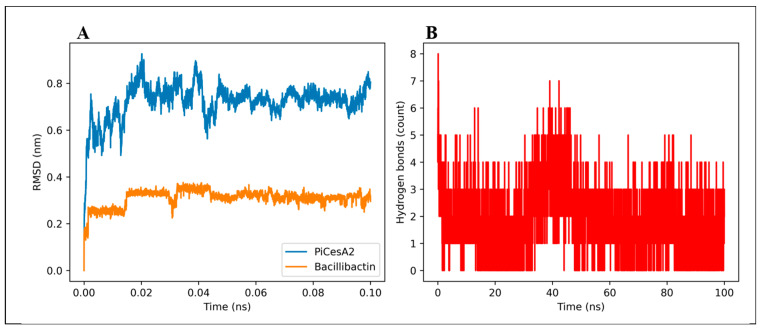
Molecular dynamics analysis of the PiCesA2–GT-A–Bacillibactin complex. (**A**) Backbone RMSD of PiCesA2 together with RMSD of the bound Bacillibactin ligand over the simulation time, indicating stable protein conformation and ligand retention. (**B**) The time evolution of protein–ligand hydrogen bonds, demonstrating persistent intermolecular interactions throughout the MD trajectory.

To evaluate the dynamic stability of these docked complexes, molecular dynamics (MD) simulations were conducted for the top-scoring ligand–protein complexes. The representative PiCesA2–Bacillibactin complex maintained stable ligand occupancy and persistent interaction with interfacial residues throughout the 100 ns simulation, as shown by restrained fluctuations in RMSD and reduced RMSF at the binding interface (Figure 7A). These results confirm the thermodynamic and conformational stability of Bacillibactin binding at the GT-A interface and support its potential role as an assembly disrupting agent.

## 4. Discussion

The biosynthesis of cellulose, a fundamental structural polysaccharide in plants and many protists, depends on the precise assembly of cellulose synthase (CesA) complexes whose multimeric organization governs both enzymatic efficiency and cell wall integrity. Cryo-EM analysis of plant CesAs demonstrated that individual CesA proteins assemble into trimers, which are further organized into higher-order rosette structures responsible for cellulose microfibril synthesis [3]. More recent biochemical work showed that different plant CesA isoforms form stable homotrimers that interact via class-specific regions (CSRs) to assemble functional CSCs [41,42,43]. Disruption of this multimeric organization selectively impairs cellulose deposition by destabilizing complex formation, demonstrating that CesA assembly is a prerequisite for functional cellulose synthase activity [44,45].

In *P. infestans*, cellulose synthesis is mediated by four CesA isoforms, among which PiCesA1, PiCesA2, and PiCesA4 function cooperatively, whereas PiCesA3 appears to act independently [7]. Genetic or chemical disruption of CesA activity severely impairs appressorium formation and pathogenicity, highlighting CesAs as critical virulence determinants [46,47,48]. In the present study, AlphaFold-Multimer was used to model the *P. infestans* CesA1–CesA2–CesA4 heterotrimer. The predicted complex displays a roughly triangular arrangement of the three CesA subunits with extensive inter-subunit contacts on the cytosolic side, reminiscent of plant CesA trimers, which also assemble into symmetric oligomeric complexes [49,50]. In both systems, each CesA protomer exhibits a comparable membrane topology consisting of a seven-helix transmembrane module coupled to a cytosolic GT-A catalytic domain, and the subunits organize into a trimeric assembly around a central axis with a compact membrane-embedded core. However, the mechanisms stabilizing the cytosolic interfaces differ markedly. In plant CesAs, trimerization is mediated primarily by the plant-conserved region (PCR), where the second α-helix and its preceding loop form a cyclic interaction network among the three subunits [50]. In this arrangement, the loop of one protomer engages the hydrophobic C-terminal segment of PCR-H2 in the adjacent protomer, and this interaction repeats sequentially to generate a triangular PCR interface that serves as the main cytosolic stabilization hub, further supported by packing interactions within the transmembrane region [50]. In contrast, the PiCesA heterotrimer lacks this PCR-driven interaction network and instead assembles through cyclic contacts between the N-terminal pleckstrin homology domains and the GT-A domains of neighboring subunits. These PH–GT interfaces are positioned on lateral surfaces distinct from the catalytic cleft, indicating that oligomerization and catalysis are structurally independent processes. This divergence suggests that while the catalytic scaffold of cellulose synthases is evolutionarily conserved, the mechanism of trimer stabilization has adapted in oomycetes through PH-domain–mediated interactions to enable heteromeric complex formation. This interpretation is consistent with the broader role of PH domains as non-catalytic interaction modules and supports their function in organizing CesA complex architecture rather than solely mediating membrane targeting [51].

The evolutionary significance of the PH domain is further underscored by its absence in both plant CesAs and PiCesA3. PH domains are ~100–130 amino-acid modules characterized by a conserved β-sandwich fold capped by a C-terminal α-helix, forming versatile platforms for protein–protein and protein–lipid interactions [52]. In many systems, clusters of basic residues located within the β1–β2 loop and adjacent strands mediate phosphoinositide binding, enabling selective recruitment of proteins to specific membrane microdomains [52]. In oomycetes, the presence of this domain in PiCesA1, PiCesA2, and PiCesA4 likely reflects a lineage-specific adaptation that facilitates cooperative assembly of heteromeric complexes. Beyond structural stabilization, the PH domain may contribute to the spatial organization of cellulose synthase complexes at the plasma membrane by promoting lipid-mediated membrane targeting and coincidence detection with other regulatory factors [53]. Such positioning could be particularly important during infection-related morphogenesis, where localized cellulose deposition is required for appressorium formation and host penetration. The lack of this domain in PiCesA3 correlates with biochemical evidence showing weaker association of this isoform with the main CesA complex, suggesting that PH-mediated interfaces are central to stable heterotrimer formation. This structural innovation may therefore provide oomycetes with additional regulatory flexibility, allowing dynamic modulation of cellulose synthesis during infection-related morphogenesis.

Although oomycete CesA3 lacks the N-terminal pleckstrin homology (PH) domain present in other oomycete CesAs, functional studies across multiple Phytophthora species demonstrate that it is essential for maintaining cell wall integrity, polarized growth, and infection structure development, implying that it can function as an independent or auxiliary synthase module [54]. Oomycete CesA3 is also the primary molecular target of carboxylic acid amide (CAA) fungicides such as mandipropamid, which inhibit cellulose biosynthesis and thereby disrupt pathogen development [54]. Resistance to these fungicides frequently arises through point mutations within CesA3, particularly in regions predicted to affect transmembrane packing or catalytic access, indicating that subtle structural changes can preserve enzymatic activity while diminishing inhibitor binding and thus limiting the durability of strategies that target single synthase subunits.

The PiCesA heterotrimeric assembly, in contrast, provides novel sites for drug design that are less easily bypassed by mutation, as disrupting inter-subunit interfaces can block formation of a functional cellulose synthase complex regardless of individual catalytic competence. Because the PH-mediated interfaces characterized in oomycete CesAs are absent from plant CesAs, these contacts constitute lineage-specific vulnerabilities that could allow selective inhibition of pathogen cellulose biosynthesis while minimizing effects on host cell wall formation, highlighting CesA assembly surfaces as promising targets for next-generation, resistance-resilient anti-oomycete strategies. A key implication of resolving the CesA heterotrimer structure is the ability to identify inhibitors that interfere with critical protein–protein interfaces rather than the catalytic site, and docking analyses have revealed several *Bacillus*-derived secondary metabolites, including Bacillibactin, as high-affinity binders of conserved lateral surfaces on the GT-A domain. *Bacillus* metabolites are increasingly recognized as effective biocontrol agents against plant pathogens, and Bacillibactin has been shown to exert direct antifungal activity beyond iron sequestration, broadening the antagonistic potential of *Bacillus* strains [55]. Together, these findings support the feasibility of exploiting *Bacillus*-derived compounds to disrupt essential oomycete cell wall machinery.

Non-catalytic inhibition of cellulose synthases has emerged as a critical strategy for achieving both specificity and durability in cellulose biosynthesis disruption. Because the UDP-glucose–binding catalytic core of CesAs is highly conserved across plants, oomycetes, and bacteria, direct targeting of this site carries a high risk of off-target effects and host toxicity. Consistent with this, several well-characterized cellulose biosynthesis inhibitors interfere with cellulose synthase complex (CSC) assembly, stability, or plasma-membrane dynamics rather than directly blocking catalysis [56]. For example, the small molecule morlin inhibits cellulose deposition by uncoupling CesA trajectories from cortical microtubules, thereby disrupting CSC guidance without impairing enzymatic activity at the active site [57]. Notably, in oomycetes, the CAA fungicide mandipropamid inhibits cellulose synthesis by targeting PiCesA3 at a site distinct from the catalytic core, demonstrating that non-catalytic CesA inhibition can be both potent and pathogen-selective [46]. In this framework, the identification of ligand-binding pockets within the PH domain and at GT-A interfacial surfaces aligns with established non-catalytic inhibition paradigms and provides a mechanistic basis for selectively disrupting oomycete CesA assembly while minimizing effects on host cellulose biosynthesis.

In conclusion, this study reframes *P. infestans* cellulose synthases as components of an obligate heteromeric assembly in which the PH domain functions as a central architectural and regulatory element. By integrating AI-based structural prediction with molecular docking, dynamics simulations, and independent biochemical evidence from Phytophthora species, we identify CesA assembly interfaces as promising targets for next-generation anti-oomycete strategies. While the present work provides mechanistic insights based on computational modeling, experimental validation, such as site-directed mutagenesis of interface residues, co-immunoprecipitation to confirm subunit interactions, and biochemical inhibitor assays, will be essential to verify the proposed assembly model and to assess its potential for developing selective and durable anti-oomycete control strategies.

## Figures and Tables

**Table 1:** Docking scores and key interacting residues of *Bacillus*-derived metabolites at the GT-A inter-subunit interface of *P. infestans* CesAs.

Compound	Target	Binding Affinity (kcal/mol)	Key Hydrogen-Bond/Polar Residues	Hydrophobic/Alkyl Residues
Bacillibactin	PiCesA1	−9.6	Tyr376, Lys394, Glu398, Arg404, Tyr425 Arg428,	Lys394, Glu398, Gly401
	PiCesA2	−9.3	Tyr385, Asp411, Arg413, Glu415, Arg437	Lys403, Glu407
	PiCesA4	−8.9	Tyr382, Ile403, Asp408, Arg410, Asp430, Arg433	Arg410, Lys434
Lipoamicoumacin B	PiCesA1	−9.2	Tyr376, Arg404, Lys429, Ser432	Leu392, Leu436
	PiCesA2	−8.6	Arg413, Tyr385, Arg437	Val396, Leu401, Leu445
	PiCesA4	−7.6	Tyr382, Asp430, Arg410	Val398, Ile403
Bacillaene	PiCesA1	−8.1	Arg428, Tyr376, Ser432	Leu392, Leu397
	PiCesA2	−8.2	Arg433, Tyr382, Asp430	Val398, Ile403
	PiCesA4	−8.4	Asp408, Arg410, Tyr382	Val398, Ile403
Leodoglucomide A	PiCesA1	−7.5	Tyr376, Arg428, Ser432	Leu392, Phe455
	PiCesA2	−7.8	Asp408, His411, Arg433	Val398, Ile403
	PiCesA4	−8.3	Tyr382, Asp430, Arg410	Val398, Val439
Gageotetrin A	PiCesA1	−7.6	Tyr376, Arg404, Ser432	Leu392, Ile390
	PiCesA2	−7.5	Tyr385, Arg437, Asp411	Leu401, Val399
	PiCesA4	−7.4	Tyr382, Arg433, Thr437	Val398, Ile403

## Data Availability

The original contributions presented in this study are included in the article/supplementary material. Further inquiries can be directed to the corresponding author.

## Supplementary Materials

The following supporting information can be downloaded at: https://www.mdpi.com/article/10.3390/jof12030192/s1, Figure S1. Per-residue predicted local distance difference test (pLDDT) scores for AlphaFold-predicted PiCesA models. Figure S2. Predicted aligned error (PAE) maps for AlphaFold-predicted PiCesA models. Figure S3. Ramachandran plot analysis of AlphaFold-predicted PiCesA models. Figure S4. Structural alignment of HADDOCK-refined PH–GT-A domain complexes with interfaces extracted from the AlphaFold-Multimer–predicted PiCesA heterotrimer. Figure S5. Multiple sequence alignment of CesA1 orthologs across representative oomycete species. Figure S6. Multiple sequence alignment of CesA2 orthologs across representative oomycete species. Figure S7. Multiple sequence alignment of CesA4 orthologs across representative oomycete species. Table S1. Details of PiCesA proteins. Table S2. Sequences used for phylogenetic analysis. Table S3. Structural quality assessment of AlphaFold-predicted PiCesA models. Table S4. HADDOCK docking statistics of PH–GT-A domain interactions. Table S5. Compounds used for docking analysis.
