# Supplementary material for: Computational Modeling of Cellulose Synthase Heterotrimer Assembly and Identification of Antimicrobial Compounds Targeting Interface Sites in Phytophthora infestans
Source: J Fungi (Basel). 2026 Mar 7;12(3):192. doi: 10.3390/jof12030192 (PMC13027454; doi:10.3390/jof12030192)
Supplement: Supplementary file 1 [file jof-12-00192-s001.zip › Supplementary Figures.pdf]

Supplementary Figures

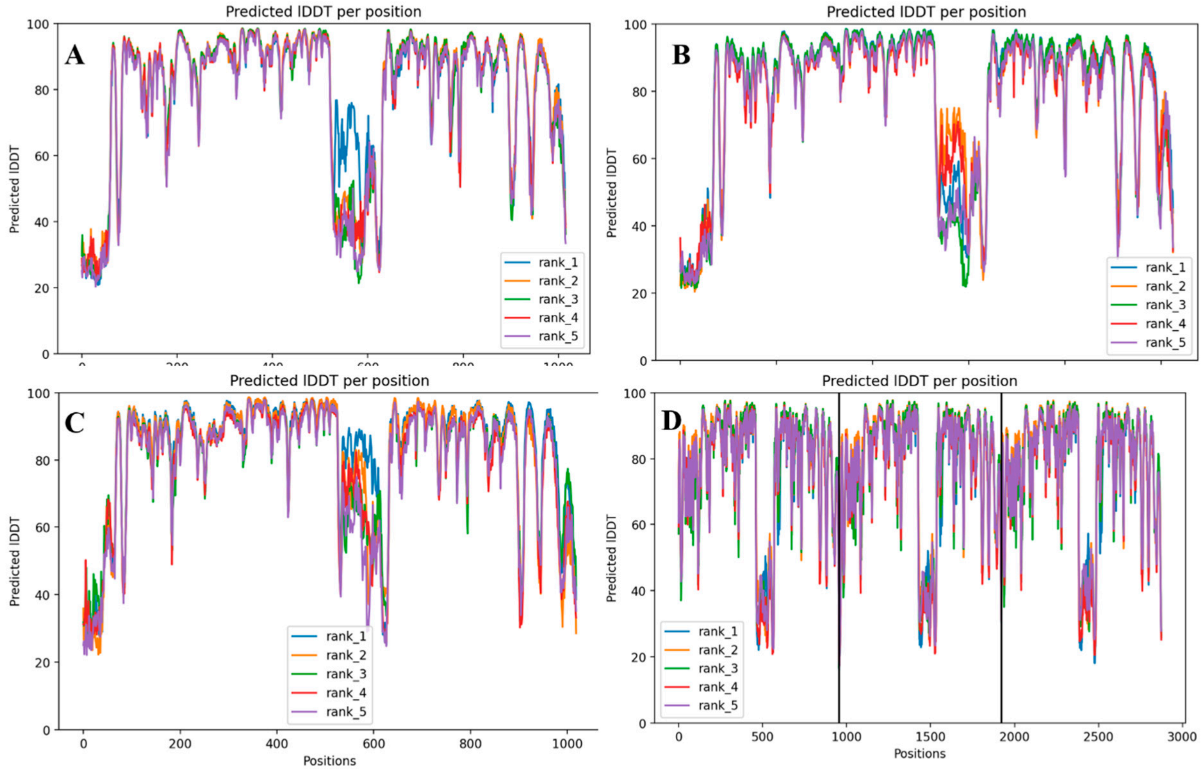

Figure S1. Per-residue predicted local distance difference test (pLDDT) scores for AlphaFold-predicted PiCesA models. Panels **A–C** show pLDDT profiles of the monomeric **PiCesA1**, **PiCesA2**, and **PiCesA4** models generated by AlphaFold3, respectively, while **D** shows the pLDDT profile of the **PiCesA1–PiCesA2–PiCesA4 heterotrimer** predicted using AlphaFold-Multimer. For each model, scores from the top five ranked predictions (rank_1–rank_5) are overlaid. High pLDDT values across most regions indicate confident structural predictions, whereas localized reductions correspond to flexible loops, terminal regions, and inter-domain linkers; vertical separators in panel D denote individual subunits within the trimeric assembly.

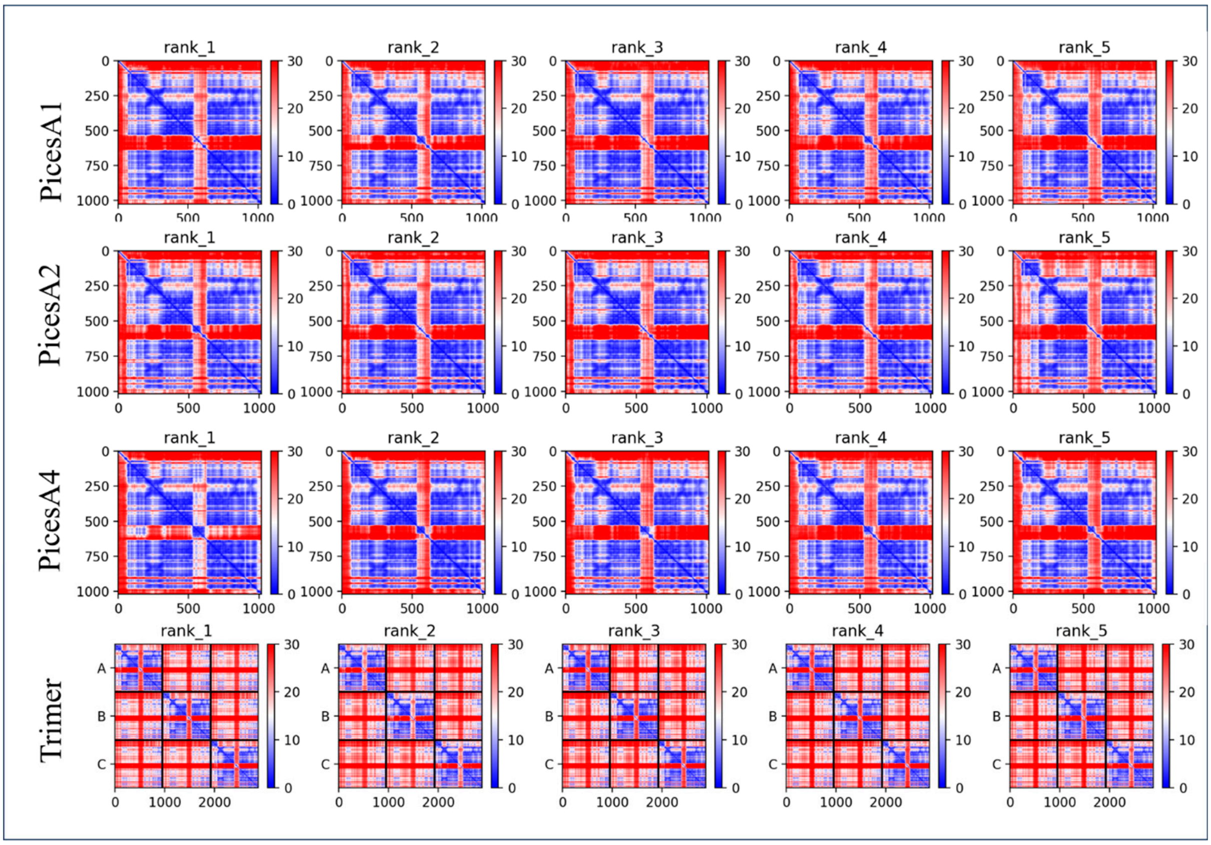

Figure S2. Predicted aligned error (PAE) maps for AlphaFold-predicted PiCesA models. Rows correspond to **PiCesA1**, **PiCesA2**, **PiCesA4** monomers (top to bottom) generated by AlphaFold3 and the **PiCesA1–PiCesA2–PiCesA4 heterotrimer** predicted using AlphaFold-Multimer (bottom row). For each model, PAE maps of the top five ranked predictions (rank_1–rank_5) are shown. Blue regions indicate low predicted alignment error (high confidence in relative residue positioning), whereas red regions denote higher uncertainty, typically associated with flexible loops or inter-domain/linker regions. In the trimeric models, block-wise low PAE along the diagonal and off-diagonal regions corresponding to subunits **A–C** indicate confident intra-subunit folding and well-defined inter-subunit interfaces, supporting the structural robustness of the predicted PiCesA heterotrimer.

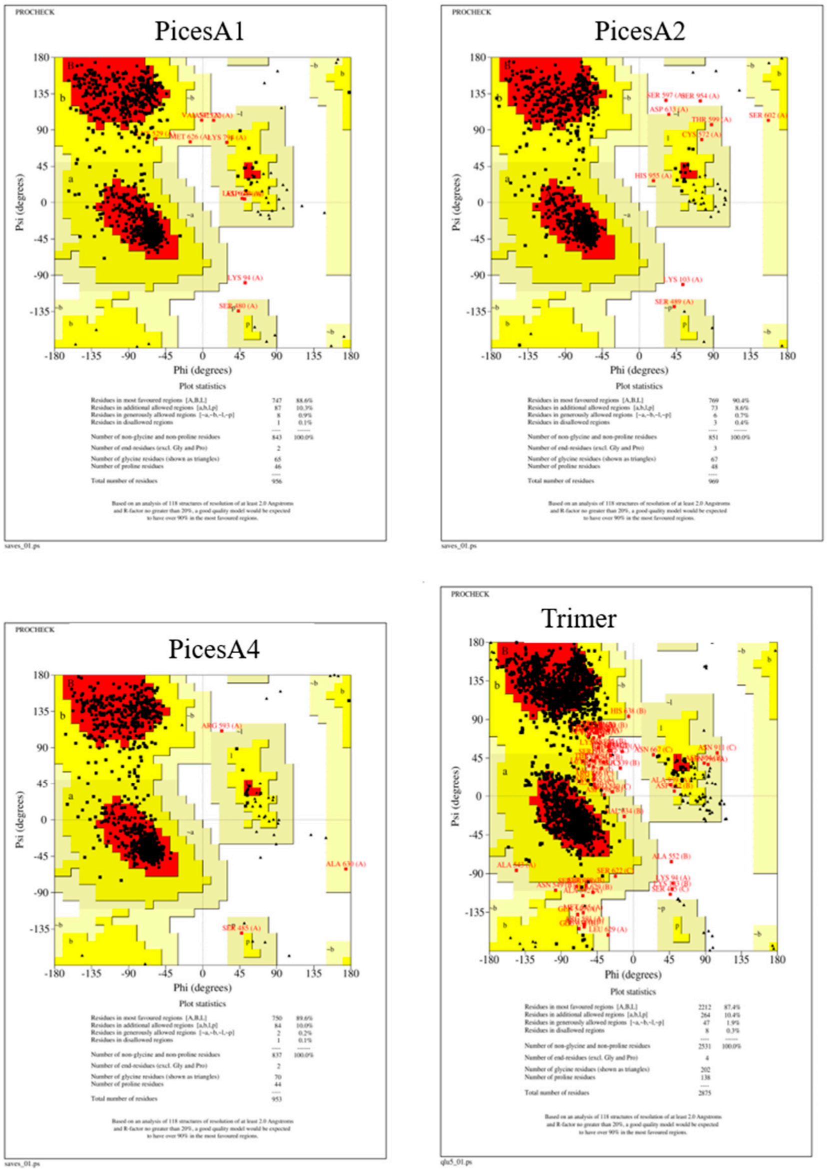

Figure S3. Ramachandran plot analysis of AlphaFold-predicted PiCesA models. Panels show PROCHECK Ramachandran plots for **PiCesA1**, **PiCesA2**, **PiCesA4** monomers and the **PiCesA1–PiCesA2–PiCesA4 heterotrimer**. Residues are distributed predominantly within the most favored (red) and additionally allowed (yellow) regions, with only a small fraction in generously allowed or disallowed regions. The plots indicate appropriate backbone φ/ψ geometry and overall stereochemical quality of both monomeric and trimeric PiCesA models, supporting their suitability for downstream structural and interaction analyses.

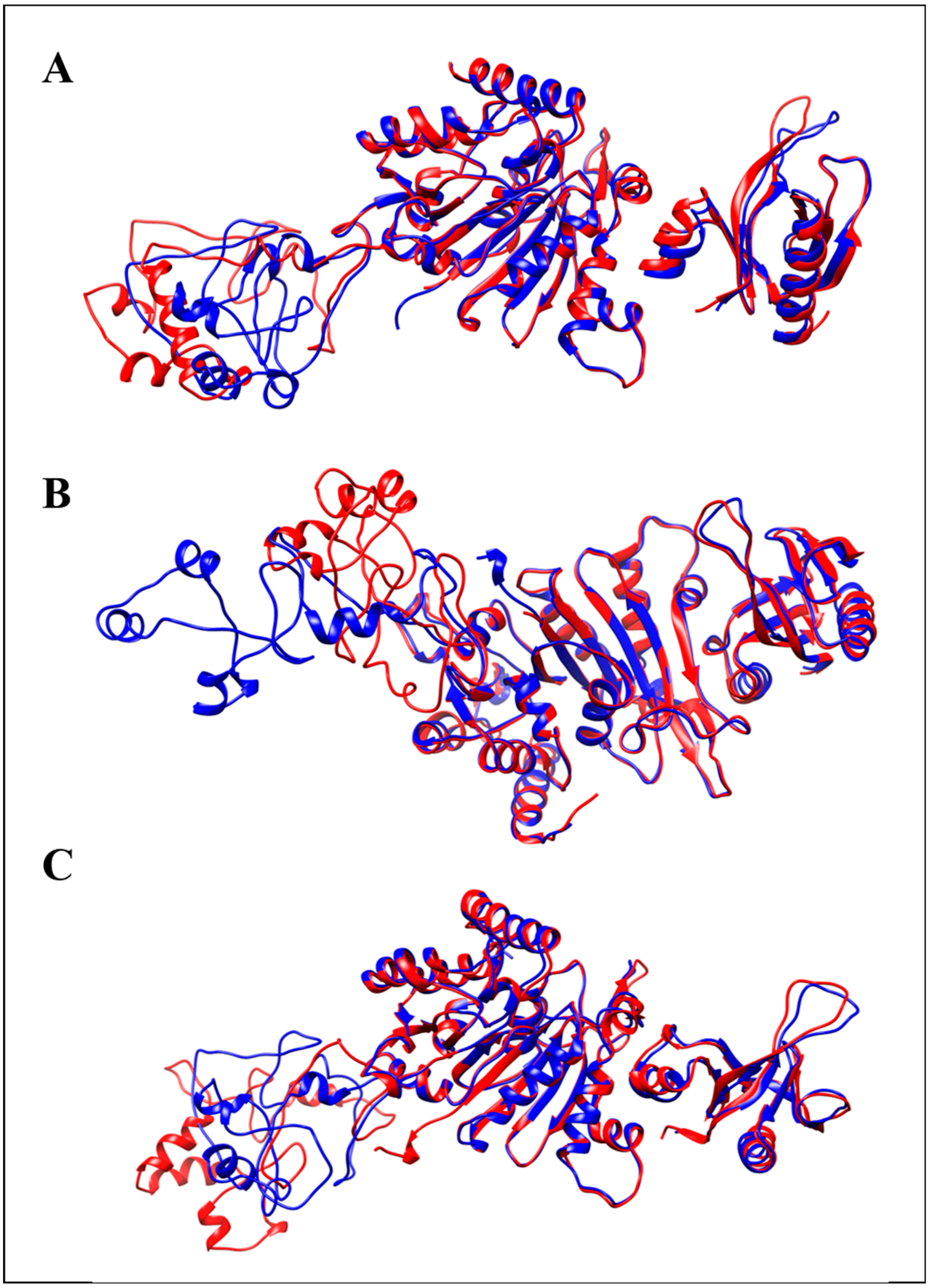

Figure S4. Structural alignment of HADDOCK-refined PH–GT-A domain complexes (blue) with the corresponding interfaces extracted from the AlphaFold-Multimer–predicted PiCesA heterotrimer (red). Superpositions demonstrate close agreement between docking and multimer predictions for **(A)** PiCesA1(PH)–PiCesA2(GT-A), **(B)** PiCesA2(PH)–PiCesA4(GT-A), and **(C)** PiCesA4(PH)–PiCesA1(GT-A). The strong overlap of secondary-structure elements supports a conserved PH–GT-A interaction geometry and independently validates the inter-subunit interfaces within the PiCesA heterotrimer.

```
                                                                                376
Phytophthora_infestans_CesA1          PDDLWPKIDVLLCHYSEPAEETIDTLMACMNLQYPPHLVQIYVLDDGYCGSKWTKGNPVP
Phytophthora_capsici_CesA1            PDDLWPKIDVLLCHYSEPAEETIDTLMACMNLQYPPHLLQIYVLDDGYCSTKWTKGNPVP
Phytophthora_cinnamomi_CesA1          PDDLWPKIDVLLCHYSEPAEETIDTLMACMNLQYPPHLLQIYVLDDGYCSTKWTKGNPIP
Phytophthora_sojae_CesA1              PDDLWPKIDVLLCHYSEPAEETIDTLMACMNLQYPPHLLQIYVLDDGYCSTKWTKGNPIP
Phytophthora_ramorum_CesA1            PDDLWPKIDVLLCHYSEPAEETIDTLMACMNLQYPPHLLQIYVLDDGYCSTKWTKGNPVP
Phytophthora_palmivora_CesA1          PDDLWPKIDVLLCHYSEPAEETIDTLMACMNLQSPPHLLQIYVLDDGYCSSKWTKGNPVP
Peronospora_effusa_CesA1              PDDLWPKIDVLLCHFSEPAEETIDTLMACMNLQYPPHLLQIYVLDDGYCSSKWTKGNPVP
Bremia_lactucae_CesA1                 PDDLWPKIDVLLCHYSESAEETIDSLLACMNLQYPPHLLQIYVLDDGYCITKWTKGSPIP
Plasmopara_viticola_CesA1             PDDLWPKIDVLLCHYSEPAEETIDTLLACINLQYPPHLLQIYVLDDGYCSTKWTKGNPVP
Globisporangium_splendens_CesA1       PDELWPKVDVFLCHYSEPAEETIDTLMACMNLQYPPHLLQIYVLDDGYCTTKWTKGNPIP
Pythium_aphanidermatum_CesA1          PDELWPKVDVLICRYTEPAEECIDTLMACMNLQYPPHLLQIYILDDGYCTTKWTKGNPVP
Pythium_insidiosum_CesA1              --------------------------------------------DDGYCTTKWTKGNPVP
                                                                            ***** :*****.*:*

                                           394  398 401 404                     425 428
Phytophthora_infestans_CesA1          AIELNKVVLEKSGDLRQEVAQFMYDRVCDPNEDME-YAWRKLHSSANLPSPSRPKVVNRA
Phytophthora_capsici_CesA1            AIELNKVVLEKSGDLRQEVAQFMYDRVCDPNEDMEVYAWRKLHSSANLPSASRPKVVNRA
Phytophthora_cinnamomi_CesA1          AIELNKVVLEKAGDLRQEVAQFMYDRVCDPNEEMEVYAWRKLHSSANLPSASRPKVVNRA
Phytophthora_sojae_CesA1              AIELNKMVLEKAGDLRQEVAQFMYDRVCDPNEEMEVYAWRKLHSSANLPSASRPKVVNRA
Phytophthora_ramorum_CesA1            AIELNKVVLEKAGDLRQEVAQFMYDRVCDPNEDMEVYAWRKLHSSANLPSASRPKVVNRA
Phytophthora_palmivora_CesA1          AIELNKMVLEKSGDLRQEVAQFMYDRVCDPNEEMEVYAWRKLHSSANLPSASRPKVVNRA
Peronospora_effusa_CesA1              AIELNKVILEKSGDLRQEVAQFMYDRVCDPNEDIEVYAWRKLHSSANLPSASRPKVVNRA
Bremia_lactucae_CesA1                 SIELNKEILEKCGDLRQEVAQFMYDRVCDPNEDIEVYAWRKLHSSANLPSASRPRVVKRA
Plasmopara_viticola_CesA1             AIELNKVILEKAGDLRQEVAQFMYDRVCDPNEDMEVYAWRKLHSSANLPSSSRPKVVNRA
Globisporangium_splendens_CesA1       AIELNKDVLEKSGDLRQEVAQFMYDRVCDPNEEMEVYAWRKLHSSANLPSPSRPKVVLRA
Pythium_aphanidermatum_CesA1          AIELYKDVLEKSGDLRQEVAQFMYDRVCDPNEEMEVYAWRKLHSSANLPSPSRPRVVNRA
Pythium_insidiosum_CesA1              AIELNKEVLEKSGDLRQEIAQFMFDRVCDPNEEMEVYAWRKLHSSANLPSPSRPRVVNRA
                                      :*** * :***.******:****:********::****************.***:** **
```

Figure S5. Multiple sequence alignment of CesA1 orthologs across representative oomycete species. Residues involved in binding of the top-ranked ligand Bacillibactin, as identified from docking analyses (Table 1), are highlighted in red. The high conservation of these residues across species supports their functional importance and suggests that the predicted binding interface represents a conserved structural feature of oomycete CesA1 proteins.

```
                                                                                     385
Phytophthora_infestans_CesA2        PDDLWPKVDVLLCHYSEPAEETIDTLMACMNLQYPPHLLQIWVCDDGYCKTKWTKGNPVP
Phytophthora_capsici_CesA2          PDELWPKVDVLLCHYSEPAEETIDTLMACMNLQYPPHLLQIWVCDDGYCKAKWTKGNPVP
Phytophthora_palmivora_CesA2        PDELWPKVDVLLCHYSEPAEETIDTLMACMNLQYPPHLLQIWVLDDGYCKAKWTKGNPVP
Plasmopara_viticola_CesA2           PDELWPKVDILLCHYSEPAEEAIDTLMACMNVQYPPHLLQIWVCDDGYCKTKWTKGNPIP
Phytophthora_ramorum_CesA2          PDELWPKVDVLLCHYSEPAEETIDTLMACMNLQYPPHLLQIWVLDDGYCKAKWAKGNPVP
Phytophthora_sojae_CesA2            PDELWPKVDVLLCHYSEPAEETIDTLMACMNLQYPPHLLQIWVCDDGYCKSKWSKGNPVP
Phytophthora_cinnamomi_CesA2        PDELWPKVDVLLCHYSEPAEETIDTLMACMNLQYPPHLLQIWVCDDGYCKSKWSKGNPVP
Peronospora_effusa_CesA2            PDELWPKVDVLLCHYSEPAEETIDTLMACMNLQYPPHLLQIWVLDDGYCKAKWAKGNPVP
Bremia_lactucae_CesA2               PDELWPKVDVLLCHYSEPAEEAIDTLMACMNLQYPPHLLQIWVCDDGYCKTKWTKGNPVP
Globisporangium_splendens_CesA2     PDELWPKVDVFLCHYSEPAEETIDTLMACMNMQYPPHLLQIYVLDDGYCKAKWQKGNPVP
Lagenidium_giganteum_CesA2          PDELWPKCDVLLCHYSEPAEECIDTLMACMNLQYPPHLLQIYILDDGYCKAKWTKGNPVP
Pythium_aphanidermatum_CesA2        PDEQWPRVDMLLCHYSEPAEECIDTLMACMNIQYPPHLLHIFILDDGYCKAKWTKGNPVP
Pythium_insidiosum_CesA2            PDEQWPRVDVLLCHYSEPAEECIDTLAACVNIQYPPHLLHIFVLDDGYCKAKWTKGNPVP
Albugo_candida_CesA2                PDELWPKVDVLICHYSEPADECVDTLNACMNLRYPPHLLHIYILDDGYCKAKWTKGNPLP
Albugo_laibachii_CesA2              PDDVWPRVDVLICHYSEPSEECIDTLSACMNLQYPPHLLQIYLLDDGYCKAKWTKENPVP
Aphanomyces_cochlioides_CesA2       PDEQWPKVDVFICHYSEPAEETIDTLAACMNLQYPPHLLQIYILDDGYCKAKWTKGNPVP
Aphanomyces_euteiches_CesA2         PDEQWPKVDVFICHYSEPAEETIDTLAACMNLQYPPHLLQIYILDDGYCKAKWAKGNPVP
Saprolegnia_parasitica_CesA2        PDEQWPKVDIFICHYSEPAEETIDTLAACMNLQYPPHLLQIYVLDDGYCKTKWTKGEEVP
Saprolegnia_diclina_CesA2           PDEQWPKVDVFICHYSEPAEETIDTLAACMNLQYPPHLLQIYVLDDGYCKTKWTKGEEVP
Saprolegnia_monoica_CesA2           PDEQWPKVDVFICHYSEPAEETIDTLAACMNLQYPPHLLQIYVLDDGYCKTNWKKGEEVP
Aphanomyces_astaci_CesA2            PDEQWPKVDVFICHYSEPAEETIDTLAACMNLQYPPHLLQIYVLDDGYCKAKWTKGNPVP
                                    **: **: *:::******::* :*** **:*::******:*:: ******::* * : :*
                                          403  407  411413415               437
Phytophthora_infestans_CesA2        TVELNKGILETAGDLRQEVAQFMYDRVCDPNEDMEVYAWRKLHSSANLPSPSRVKVVNRA
Phytophthora_capsici_CesA2          TVELNKGILETAGDLRQEVAQFMYDRVCDPNEDMEVYAWRKLHSSANLPSPSRVKVVNRA
Phytophthora_palmivora_CesA2        TVELNKGILETAGDLRQEVAQFMYDRVCDPNEEMEVYAWRKLHSSANLPSPSRPKVVNRA
Plasmopara_viticola_CesA2           TVELNKGILETAGDLRQEVAQFMYDRVCDPNEDMEVYAWRKLHSSANLPSPSRCKVVNRA
Phytophthora_ramorum_CesA2          TVELNKGILETAGDLRQEVAQFMYDRVCDPNEDMEVYAWRKLHSSANLPSPSRVKVVNRL
Phytophthora_sojae_CesA2            TVELNKGILETAGDLRQEVAQFMYDRVCDPNEEMEVYAWRKLHSSANLPSPSRPRVVNRL
Phytophthora_cinnamomi_CesA2        TVELNKGILETAGDLRQEVAQFMYDRVCDPNEEMEVYAWRKLHSSANLPSPSRPKVVNRL
Peronospora_effusa_CesA2            TVELNKGILETAGDLRQEVAQFMYDRVCDPNEDTEVYAWRKLHSSANLPSPSRSKAVNRA
Bremia_lactucae_CesA2               TVELNKGVLETAGDLRQEVAQFMYDRVCDPSEEMEVYAWRKLHSSANLPSPSRVKVVNRA
Globisporangium_splendens_CesA2     TVELNKGILETAGDLRQEVAQFMYDRVCDPNEEMEVYAWRKLHSSANLPSASRPKVVNRV
Lagenidium_giganteum_CesA2          AIELNKGILETAGDLRQEVAQFMYDRVCDPNEEMEVYQWRKLHSSANLPSPSRPKVVNRL
Pythium_aphanidermatum_CesA2        QIELNKGILETAGDLRQEVAQFMYDRVCDPNEEMEVYQWRKLHSSANLPSPSRPKVVNRA
Pythium_insidiosum_CesA2            QIELNKGILETAGDLRQEVAQFMYDRVCDPNEEMEVYQWRKLHSSANLPSPSRPKVVNRA
Albugo_candida_CesA2                KIELNKGVLESAGDLRQEVAQFMYDRVCDVNEEMEVYAWRKLHSSANLPSASRPRVVDRA
Albugo_laibachii_CesA2              TIELNKGVLEAAGDLRQEVAQFMYDRVCDPAEEMEVYAWRKLHSSANIPTPSRPKAVNRS
Aphanomyces_cochlioides_CesA2       TIELNKAILESAGDLRQEVAQFMYDRVCDPNEEMEVYAWRKLHSSANLPTASRTRVVNRA
Aphanomyces_euteiches_CesA2         TIELNKGILESAGDLRQEVAQFMYDRVCDPNEEMEVYAWRKLHSSANLPTASRTRVVNRA
Saprolegnia_parasitica_CesA2        TIELNKGVLESAGDLRQEIAQFMYDRVCDPNEDMEVYAWRKLHSSANLPTPSRPRVVNRA
Saprolegnia_diclina_CesA2           TIELNKGVLESAGDLRQEIAQFMYDRVCDPNEDMEVYAWRKLHSSANLPTPSRPRVVNRA
Saprolegnia_monoica_CesA2           TIELNKSILESAGDLRQEIAQFMYDRVCDPNEDMEVYAWRKLHSSANLPTPSRPRVVNRA
Aphanomyces_astaci_CesA2            TIELNKGVLESAGDLRQEVAQFMYDRVCDPNEDMEVYAWRKLHSAANLPTASRPRVVNRA
                                     :****.:**:*******:**********  *: *** ******:**:*:.** :.*:*
```

Figure S6. Multiple sequence alignment of CesA2 orthologs across representative oomycete species. Residues involved in binding of the top-ranked ligand Bacillibactin, as identified from docking analyses (Table 1), are highlighted in red. The high conservation of these residues across species supports their functional importance and suggests that the predicted binding interface represents a conserved structural feature of oomycete CesA2 proteins.

```
                                                                                    382
Bremia_lactucae_CesA4                PPEKWPTVDILLCHYSEPAEDTIATLEKIMSLDYPPHLYHVWICDDGYCKAAWSAGAQVP
Peronospora_effusa_CesA4             PPEKWPTVDIMLCHYAEPADDTIATLEKIMNLDYPPHLFHVWICDDGYCKSKWEPGSQVP
Phytophthora_capsici_CesA4           PPEKWPTVDILLCHYAEPAEDTIATLEKIMNLDYPPHLFHVWICDDGYCKSKWEAGAQVP
Phytophthora_cinnamomi_CesA4         PPEKWPTVDILLCHYAEPAEDTIATLERIMNLDYPPHLFHVWICDDGYCKSKWEAGANVP
Phytophthora_sojae_CesA4             PPEKWPTVDILLCHYAEPAEDTIATLEKIMNLDYPPHLFHVWICDDGYCKSKWEPGATVP
Phytophthora_ramorum_CesA4           PPEKWPTVDIMLCHYAEPAEDTIATLEKIMNLDYPPHLFHVWICDDGYCKAKWEAGAQVP
Phytophthora_infestans_CesA4         PPEKWPTVDILLCHYAEPADDTIATLEKIMNLDYPPHLFHVWICDDGYCKAKWEAGATVP
Plasmopara_viticola_CesA4            PPSKWPTVDILLCHYAEPAEDTIATLEKIMNLDYPPHLVHVWICDDGYCKAKWEPGAPVP
Globisporangium_splendens_CesA4      PPSKWPTVDILLCHYSEPAEDTIATLEKIMNLDYPPHLFHVWICDDGYCKTKWEPGATIP
Pythium_aphanidermatum_CesA4         PPSKWPTVDILLCHYAEPAEDTIATLEKIMALDYPPHLYHIWICDDGYCKSKWEPGASVP
Pythium_insidiosum_CesA4             PPSKWPTVDILLCHYAEPAEDTIATLEKIMNLDYPPHLFNVWICDDGYCKSKWDDGATVP
Lagenidium_giganteum_CesA4           PPAKWPTVDILLCHYSEPAEDTIATLEKIMNLNYPPQLFHVWICDDGYCKSKWDEGASVP
Albugo_candida_CesA4                 -PSKWPPVDILLCHYAEPAEDTIATLEKIMEIDYPSHLVHVWICDDGYCKSKWK-NDPVP
Albugo_laibachii_CesA4               PPSKWPTVDILLCHYSEPAEDTIATLDKIMEVDYPSHLMHVWICDDGYCKAKWK-NDPVP
Aphanomyces_cochlioides_CesA4        PSDQWPTVDILLCHYAEPAEDTMATMEAIMKLDYPCEKLHVYICDDGGFKTKFTPGNPIP
Aphanomyces_euteiches_CesA4          PSDQWPTVDILLCHYAEPAEDTMATMEAIMKLDYPCEKLHVYICDDGGFKTKFTPGNPIP
Saprolegnia_diclina_CesA4            PSDKWPSVDILLCHYAEPAEDTMATLDAILKLDYPPEKLHVYICDDGAFKTKFTPGNPIP
                                      . :**.***:****:***:**:**:: *: ::** .  :::*****  *: :  .  :*

                                            403  408 410,                          430 433
Bremia_lactucae_CesA4                NVSVNHGVIENAGDVRHEVAQFMYDRVCESY------------ELEVDEWRKEHTTVKMP
Peronospora_effusa_CesA4             KVSVNTSVIEDAGDVRHEVAQFMYDRVCESY------------ELEVDEWRKEHTTVKMP
Phytophthora_capsici_CesA4           KVSVNTGVIEEAGDVRHEVAQFMYDRVCESY------------ELEVDEWRKEHTTVKMP
Phytophthora_cinnamomi_CesA4         KVSVNYGVIEDAGDVRHEVAQFMYDRVCESY------------ELEVDEWRKEHTTVKMP
Phytophthora_sojae_CesA4             KVSVNYGVIEDAGDVRHEVAQFMYDRVCESY------------ELEVDEWRKEHTTVKMP
Phytophthora_ramorum_CesA4           KVSVNYGVIEEAGDVRHEVAQFMYDRVCESY------------ELEVDEWRKEHTTVKMP
Phytophthora_infestans_CesA4         KVSVNTGVIEDAGDVRHEVAQFMYDRVCESY------------ELEVDEWRKEHTTVKMP
Plasmopara_viticola_CesA4            KVSVNHGVIEDAGDVRHEVAQFMYDRVCESY------------ELEVDEWRKEHTTVKMP
Globisporangium_splendens_CesA4      KVGVNRGLIEDAGDVRHEVAQFMYERVYESD------------EVEVDEWRKQHTTVKMP
Pythium_aphanidermatum_CesA4         KVGINRGVIEDAGDVRREVAQFMFERVCESD------------DIEVDEWRKQHTHVKMP
Pythium_insidiosum_CesA4             KVSINHGVIENAGDVRREVAQFMYERVCESD------------DMEVDEWRKQHTTVKMP
Lagenidium_giganteum_CesA4           KVGVNKGVIENAGDVRREVAQFMFERVCESD------------DIEVDEWRKQHTTVKMP
Albugo_candida_CesA4                 QVSVNRGVIENAGDVRREVAQFMYERVCESD------------DMEVEEWRKQHTTVKMP
Albugo_laibachii_CesA4               QVSVNRGVIENAGDVRREVAQFMYERVCESD------------DIEVEEWRKQHTTVKMP
Aphanomyces_cochlioides_CesA4        EVFVNKGVIENAGDVRYELAHFMYERVLDLDGEGQGIGYTRAVEMEVNEWRQSHTTVKMP
Aphanomyces_euteiches_CesA4          EVFVNKGVIENAGDVRYELAHFMYERVLDLDGEGQGIGYTRAVEMEVNEWRQSHTTVKMP
Saprolegnia_diclina_CesA4            DVIVNQGVIENAGDVRYELAHFMYEKVLDLDGDGQGIGYTRAAEMEVNEWRRSHTTVKLP
                                     .* :*  .:**:***** *:*:**:::* :               ::**:***:.** **:*
```

Figure S7. Multiple sequence alignment of CesA4 orthologs across representative oomycete species. Residues involved in binding of the top-ranked ligand Bacillibactin, as identified from docking analyses (Table 1), are highlighted in red. The high conservation of these residues across species supports their functional importance and suggests that the predicted binding interface represents a conserved structural feature of oomycete CesA4 proteins.
