# Supplementary material for: Computational Modeling of Cellulose Synthase Heterotrimer Assembly and Identification of Antimicrobial Compounds Targeting Interface Sites in Phytophthora infestans
Source: J Fungi (Basel). 2026 Mar 7;12(3):192. doi: 10.3390/jof12030192 (PMC13027454; doi:10.3390/jof12030192)
Supplement: Supplementary file 1 [file jof-12-00192-s001.zip › Supplementary Tables.pdf]

Table S1. Details of PicesA proteins

protein	Total length (AA)	Uniprot id	PH Domain position	GTA Domain position	Substrate binding residues
PiCesA1	1,016	ABP96902	67-175	334-769	D505, D503
PicesA2	1,026	ABP96903	75-176	343-777	D512, D514
PiCesA3	1142	ABP96904	Absent	546-886	D717, D719
PiCesA4	1,019	ABP96905	69-176	340-770	D508, D510

Table S2. List of Sequences used for the phylogenetic analysis

Kingdom	Species	CesA_isoform	Accession_or_GeneID
Oomycete	Phytophthora infestans T30-4	CesA1	XP_002902506.1
Oomycete	Phytophthora capsici	CesA1	KAG1696447.1
Oomycete	Phytophthora cinnamomi	CesA1	XP_067790066.1
Oomycete	Phytophthora ramorum	CesA1	ABP96910.1
Oomycete	Phytophthora palmivora	CesA1	POM80620.1
Oomycete	Phytophthora sojae	CesA1	XP_009524466.1
Oomycete	Peronospora effusa	CesA1	RMX64215.1
Oomycete	Plasmopara viticola	CesA1	ADD84670.1
Oomycete	Bremia lactucae	CesA1	XP_067822182.1
Oomycete	Globisporangium splendens	CesA1	KAF1335882.1
Oomycete	Lagenidium giganteum	CesA1	DBA03012.1
Oomycete	Pythium aphanidermatum	CesA1	AEZ51037.1
Oomycete	Pythium insidiosum	CesA1	KAJ0395879.1
Oomycete	Albugo candida	CesA1	CCI39601.1
Oomycete	Albugo laibachii Nc14	CesA1	CCA27796.1
Oomycete	Saprolegnia parasitica CBS 223.65	CesA1	XP_012200800.1
Oomycete	Saprolegnia diclina VS20	CesA1	XP_008620166.1
Oomycete	Saprolegnia monoica	CesA1	ACX56230.1
Oomycete	Phytophthora infestans T30-4	CesA2	KAI9993248.1
Oomycete	Phytophthora capsici	CesA2	KAG1696448.1
Oomycete	Phytophthora cinnamomi	CesA2	XP_067789926.1
Oomycete	Phytophthora ramorum	CesA2	XP_067749360.1
Oomycete	Phytophthora palmivora	CesA2	POM73223.1
Oomycete	Phytophthora sojae	CesA2	XP_009524465.1
Oomycete	Plasmopara viticola	CesA2	ADD84671.1
Oomycete	Peronospora effusa	CesA2	RMX64214.1
Oomycete	Bremia lactucae	CesA2	XP_067822182.1
Oomycete	Globisporangium splendens	CesA2	KAF1335884.1
Oomycete	Lagenidium giganteum	CesA2	DBA03012.1
Oomycete	Pythium aphanidermatum	CesA2	AEZ51038.1
Oomycete	Pythium insidiosum	CesA2	GLE03066.1
Oomycete	Albugo candida	CesA2	KAL0585899.1
Oomycete	Albugo laibachii Nc14	CesA2	CCA27796.1
Oomycete	Aphanomyces cochlioides	CesA2	KAG9403494.1
Oomycete	Aphanomyces euteiches	CesA2	KAH9162205.1
Oomycete	Aphanomyces astaci	CesA2	RHY95033.1
Oomycete	Saprolegnia parasitica CBS 223.65	CesA2	XP_012200800.1
Oomycete	Saprolegnia diclina VS20	CesA2	XP_008620166.1
Oomycete	Saprolegnia monoica	CesA2	ACX56230.1
Oomycete	Phytophthora infestans T30-4	CesA3	ABP96904.1
Oomycete	Phytophthora aleatoria	CesA3	KAG6953482.1
Oomycete	Phytophthora palmivora	CesA3	AFB20355.1
Oomycete	Phytophthora capsici	CesA3	AFB20353.1
Oomycete	Plasmopara viticola	CesA3	ADD84672.1
Oomycete	Plasmopara halstedii	CesA3	KAL7682188.1
Oomycete	Bremia lactucae	CesA3	AFB20351.1
Oomycete	Pseudoperonospora cubensis	CesA3	AEC45570.1

Oomycete	Hyaloperonospora brassicae	CesA3	CAI5741748.1
Oomycete	Globisporangium violae	CesA3	AFB20360.1
Oomycete	Globisporangium irregulare	CesA3	AFB20358.1
Oomycete	Globisporangium iwayamae	CesA3	AFB20359.1
Oomycete	Globisporangium splendens	CesA3	KAF1319076.1
Oomycete	Lagenidium giganteum	CesA3	AIK98134.1
Oomycete	Pythium aphanidermatum	CesA3	AEZ51039.1
Oomycete	Pythium coloratum	CesA3	AFB20357.1
Oomycete	Pythium oligandrum	CesA3	TMW66986.1
Oomycete	Pythium insidiosum	CesA3	KAJ0407184.1
Oomycete	Pythium arrhenomanes	CesA3	AFB20356.1
Oomycete	Albugo candida	CesA3	KAL0585507.1
Oomycete	Albugo laibachii Nc14	CesA3	CCA23182.1
Oomycete	Aphanomyces euteiches	CesA3	CAK4494417.1
Oomycete	Aphanomyces cochlioides	CesA3	KAG9413046.1
Oomycete	Aphanomyces invadans	CesA3	XP_008861121.1
Oomycete	Aphanomyces astaci	CesA3	RHY24164.1
Oomycete	Aphanomyces stellatus	CesA3	KAF0690348.1
Oomycete	Saprolegnia parasitica CBS 223.65	CesA3	XP_012200166.1
Oomycete	Saprolegnia diclina VS20	CesA3	XP_008617056.1
Oomycete	Saprolegnia monoica	CesA3	ACX56231.1
Oomycete	Phytophthora infestans T30-4	CesA4	XP_002897200.1
Oomycete	Phytophthora capsici	CesA4	KAG1695095.1
Oomycete	Phytophthora cinnamomi	CesA4	XP_067782607.1
Oomycete	Phytophthora ramorum	CesA4	ABP96913.1
Oomycete	Phytophthora sojae	CesA4	XP_009534094.1
Oomycete	Plasmopara viticola	CesA4	ADD84673.1
Oomycete	Peronospora effusa	CesA4	CAI5713359.1
Oomycete	Bremia lactucae	CesA4	XP_067819866.1
Oomycete	Globisporangium splendens	CesA4	KAF1315159.1
Oomycete	Lagenidium giganteum	CesA4	DAZ93465.1
Oomycete	Pythium aphanidermatum	CesA4	AEZ51040.1
Oomycete	Pythium insidiosum	CesA4	GLD98252.1
Oomycete	Albugo candida	CesA4	CCI47178.1
Oomycete	Albugo laibachii Nc14	CesA4	CCA19369.1
Oomycete	Aphanomyces cochlioides	CesA4	KAG9404378.1
Oomycete	Aphanomyces euteiches	CesA4	KAF0727680.1
Oomycete	Saprolegnia diclina VS20	CesA4	XP_008614905.1
Plant	Solanum tuberosum	CesA1	STCesA1
Plant	Solanum tuberosum	CesA2	STCesA2
Plant	Solanum tuberosum	CesA3	STCESA3
Plant	Solanum tuberosum	CesA4	STCESA4
Plant	Solanum tuberosum	CesA5	STCESA5
Plant	Solanum tuberosum	CesA6	STCESA6
Plant	Solanum tuberosum	CesA7	STCESA7
Plant	Solanum tuberosum	CesA8	STCESA8
Plant	Solanum lycopersicum	CesA1	SLCesA1
Plant	Solanum lycopersicum	CesA2	SLCesA2
Plant	Solanum lycopersicum	CesA3	SLCESA3
Plant	Solanum lycopersicum	CesA4	SLCESA4

Plant	Solanum lycopersicum	CesA5	SLCESA5
Plant	Solanum lycopersicum	CesA6	SLCESA6
Plant	Solanum lycopersicum	CesA7	SLCESA7
Plant	Solanum lycopersicum	CesA8	SLCESA8

Table S3. Structural quality assessment and domain organization of AlphaFold-predicted PiCesA models

Protein / Assembly	Total length (aa)	pLDDT confidence (overall)	PAE within domains	ERRAT2 score (%)	Ramachandran favored (%)	Ramachandran additionally allowed (%)	Ramachandran disallowed (%)
PiCesA1	1016	High (>85)	Low	96.19	88.6	10.9	0.5
PiCesA2	1026	High (>85)	Low	95.15	90.4	8.7	0.9
PiCesA4	1019	Very high (>88-90)	Low	93.52	89.6	10.2	0.2
PicesA-trimer	3061	High (>80)	Low within subunits		87.4	11.8	0.8

Table S4. HADDOCK docking statistics summarizing energetics and interface properties of PH–GT-A interactions among PiCesA subunits.

**Docking Pair**	**Interacting Domains**	**Active Residues (PH domain)**	**Active Residues (GT-A domain)**	**HADDOCK Score (a.u.)**	**van der Waals Energy (kcal·mol^{-1})**	**Electrostatic Energy (kcal·mol^{-1})**	**Desolvation Energy (kcal·mol^{-1})**	**Buried Surface Area (Å^2)**	**Cluster Size**	**RMSD from AF-Multimer (Å)**
PiCesA1–PiCesA2	PH (PiCesA1) – GT-A (PiCesA2)	Arg107, Glu105, Arg113, Asn61, Asp116	Glu357, Glu415, Glu432, Lys403, Arg437	−112.4 ± 6.3	−68.1 ± 4.2	−285.7 ± 15.6	−24.3 ± 2.1	1,850 ± 120	42	1.9
PiCesA2–PiCesA4	PH (PiCesA2) – GT-A (PiCesA4)	Lys83, Tyr110, Asp125, Gln117, Asn123	Arg410, Arg433, Glu435, Tyr382	−108.7 ± 7.1	−64.9 ± 5.1	−271.3 ± 18.4	−22.8 ± 2.4	1,720 ± 140	38	2.7
PiCesA4–PiCesA1	PH (PiCesA4) – GT-A (PiCesA1)	Arg113, Asn117, Ser118, Asp111	Glu423, Arg428, Lys394, Tyr376	−104.2 ± 5.8	−61.3 ± 3.9	−259.6 ± 14.8	−21.1 ± 1.9	1,640 ± 110	35	2.3

Table S5. List of compounds used in the docking analysis

No.	Metabolite Name	Bacillus Species	PubChem ID
1	Macrolactin A	Bacillus amyloliquefaciens	10949443
2	Macrolactin D	Bacillus amyloliquefaciens	146684980
3	Amylocyclicin	Bacillus amyloliquefaciens	24610713
4	Macrolactin W	Bacillus amyloliquefaciens	54581687
5	Bacillomycin F	Bacillus amyloliquefaciens	3086138
6	Bacillomycin D	Bacillus amyloliquefaciens	139585475
7	Plantazolicin	Bacillus amyloliquefaciens	101802948
8	Difficidin	Bacillus amyloliquefaciens	125281
9	Bacillomycin L	Bacillus amyloliquefaciens	135321116
10	Oxydifficidin	Bacillus amyloliquefaciens	196326
11	Daptomycin	Bacillus amyloliquefaciens	71311849
12	Gramicidin D	Bacillus brevis	45267103
13	Gramicidin S	Bacillus brevis	73357
14	Cereulide	Bacillus cereus	10057089
15	Gageotetrin	Bacillus cereus	102225143
16	Zwittermicin A	Bacillus cereus	21120914
17	Butirosin A	Bacillus circulans	12302171
18	Lichenysin D	Bacillus licheniformis	177596772
19	Bacitracin A	Bacillus licheniformis	10909430
20	Bacitracin C	Bacillus licheniformis	172866290
21	Lichenysin A	Bacillus licheniformis	122233594
22	Ieodoglucomide A	Bacillus licheniformis	57342098
23	Polymyxin B	Bacillus polymyxa	49800004
24	Pseudomycin	Bacillus polymyxa	139587277
25	Fusaricidin	Bacillus polymyxa	139586954
26	Pumilacidin A	Bacillus pumilus	156581858

27	Pumilacidin B	Bacillus pumilus	21776509
28	Bacillamide B	Bacillus sp.	24178902
29	Bacillamide A	Bacillus sp.	11077881
30	Lipoamicoumacin B	Bacillus subtilis	101884392
31	Bacilosarcin A	Bacillus subtilis	24905973
32	Bacillibactin	Bacillus subtilis	125349
33	Bacilysin	Bacillus subtilis	86583338
34	Bacillithiol	Bacillus subtilis	42614123
35	Entianin	Bacillus subtilis	122216491
36	Fengycin A	Bacillus subtilis	177857407
37	Surfactin C14	Bacillus subtilis	44227776
38	Lantibiotic B	Bacillus subtilis	75237168
39	Surfactin A2	Bacillus subtilis	171390218
40	Bacillistatin	Bacillus subtilis	45268830
41	Rhizocticin	Bacillus subtilis	46174006
42	Fengycin C	Bacillus subtilis	5491283
43	Herbicolin	Bacillus subtilis	139589111
44	Iturin A	Bacillus subtilis	102287549
45	Subtilin	Bacillus subtilis	16129737
46	Mycosubtilin	Bacillus subtilis	3083700
47	Subtilosin A	Bacillus subtilis	16132390
48	Bacillaene	Bacillus subtilis	25144999
49	Amicoumacin C	Bacillus subtilis	11825660
50	Thuricin	Bacillus thuringiensis	139584544
51	Kurstakin	Bacillus thuringiensis	139584888
52	Paenilarvins	Paenibacillus larvae	139588780
53	Paenibacterin	Paenibacillus polymyxa	78319277
54	Xantholysin	Pseudomonas associated Bacillus	145720590
